# Wnt/β-catenin pathway is a key signaling pathway to trastuzumab resistance in gastric cancer cells

**DOI:** 10.1186/s12885-023-11447-4

**Published:** 2023-09-29

**Authors:** Yuna Kim, Yoo Jin Bae, Jie-Hyun Kim, Hyunki Kim, Su-Jin Shin, Da Hyun Jung, Hyojin Park

**Affiliations:** 1 Department of Internal Medicine, Division of Gastroenterology, Gangnam Severance Hospital, Yonsei University College of Medicine, 20, Eonju-ro 63-gil, Gangnam-gu, Seoul, 06229 Korea; 2 Department of Pathology, Yonsei University College of Medicine, Seoul, 03722 Korea; 3 Department of Pathology, Gangnam Severance Hospital, Yonsei University College of Medicine, Seoul, 06229 Korea; 4 Department of Internal Medicine, Division of Gastroenterology, Yonsei University College of Medicine, Seoul, 03722 Korea

**Keywords:** Wnt, Gastric cancer, Trastuzumab, Resistance, Epithelial to mesenchymal transition

## Abstract

**Background:**

Trastuzumab is the only approved target agent for the first-line treatment of human epidermal growth factor receptor-2 (HER-2) positive gastric cancer; however, trastuzumab resistance is a major problem in clinical practice. To comprehend the mechanism of trastuzumab resistance, we focused on the Wnt/β-catenin signaling pathway and its influence on the phenotypes and behavior of trastuzumab-resistant gastric cancer cells.

**Methods:**

Trastuzumab-resistant NCI-N87R cells were established in vitro from the human gastric cancer cell line NCI-N87 by dose-escalating repeated trastuzumab treatment. We investigated the phenotypes of NCI-N87R cells, including Wnt signaling pathway activity. Gastric cancer organoid cells were incubated with complete medium and Wnt3a-depletion medium, and their resistance to trastuzumab was compared.

**Results:**

NCI-N87R exhibited stemness and epithelial-mesenchymal transition (EMT)-like phenotypes, along with decreased levels of the epithelial marker E-cadherin and increased levels of the mesenchymal markers Vimentin and Snail along with an increased Wnt signaling pathway activity. When gastric cancer cells were incubated in Wnt3a-conditioned medium. Wnt signaling pathway activity and resistance to trastuzumab increased. Gastric cancer patient-derived organoids incubated in Wnt3a-depletion medium were more susceptible to dose-dependent inhibition of cell viability by trastuzumab than those incubated in complete medium.

**Conclusions:**

Trastuzumab-resistant gastric cancer cells exhibited EMT-like phenotype, and trastuzumab resistance was promoted by the Wnt/β-catenin signaling pathway. The Wnt/β-catenin pathway is a key signaling pathway for trastuzumab resistance in gastric cancer cells.

**Supplementary Information:**

The online version contains supplementary material available at 10.1186/s12885-023-11447-4.

## Introduction

Gastric cancer ranks fifth in incidence and fourth in mortality globally, with an estimate of one million new cases and 769,000 deaths reported in 2020 [1]. Human epidermal growth factor receptor-2 (HER-2), a member of the epidermal growth factor receptor (EGFR) family, is an important treatment target for gastric cancer [2]. Amplification of the HER2 gene is observed in approximately 20% of patients with gastric cancer [3, 4]. Previous studies have suggested that HER-2 overexpression is positively associated with cancer cell proliferation, malignancy, metastasis, and unfavorable outcomes [2, 5, 6]. HER-2 overexpression has also been observed in other solid tumors, including biliary tract, colorectal, non-small-cell lung, and bladder cancers [7–10]. Treatment with trastuzumab, an anti HER-2 antibody, has substantially increased the overall survival rate of patients with HER2-overexpressing cancers; however, trastuzumab resistance develops in most patients within a year [11–13]. Although new agents have been investigated to delay the onset of resistance, the duration of response to trastuzumab is limited by acquired resistance [14]. Thus, it is necessary to characterize the resistance mechanism of trastuzumab to provide alternative treatment options for patients that will inevitably develop resistance to the drug.

Numerous factors, including loss of phosphatase and the tensin homolog gene (PTEN) function [15], mutation of the phosphatidylinositide 3-kinase (PI3K)/AKT pathway [16], upregulation of insulin-like growth factor receptor (IGFR) and hetero-dimerization of IGFR/HER-20 [17], and accumulation of truncated HER-2 receptors (p95HER-2) have been found to be involved in trastuzumab resistance [18]. Previous studies suggested that prolonged treatment of tumor cells with chemotherapeutic drugs in vitro enriched the cell population with cancer stem cell (CSC) characteristics, such as high clonogenicity, expression of stemness-related genes, self-renewal ability, and resistance to chemotherapy [19]. Through epithelial to mesenchymal transition (EMT), CSCs can emerge from differentiated cancer cells. The significance of EMT in metastasis, tumor invasion, and drug resistance has become apparent [20, 21]. Moreover, EMT-like transition is known to be regulated by EMT signaling pathways, such as Wnt, Notch, and Hedgehog [16].

In a previous study, we demonstrated that HER-2 overexpressing gastric cancer cells exhibit stemness and an EMT-like phenotype, which is mediated by the Wnt/β-catenin signaling pathway [22]. Therefore, we focused on the Wnt signaling pathway and its influence on the phenotypes and behaviors of trastuzumab-resistant gastric cancer cells. This study aimed to elucidate the mechanisms underlying trastuzumab resistance in HER2-overexpressing gastric cancer.

## Materials and methods

### Gastric cancer cell line and culture

Gastric cancer cell lines including MKN45, MKN74, SNU216, SNU484, NCI-N87, and AGS were obtained from and authenticated by the Korean Cell Line Bank with STR profiling. All cells were cultured in the RPMI 1640 medium that had 10% FBS added to it at 37 °C and 5% CO_2_ in a humid incubator.

### Preparation of conditioned medium

Wnt3a-conditioned medium was harvested from Wnt3a expressing L-cells (CRL-2647; American Type Culture Collection, Manassas, VA) according to the manufacturer’s instructions. Wnt3a-conditioned medium was depleted of the Wnt3a protein by incubation with 4 µg/ml rabbit anti-Wnt3a antibody (#2391; Cell Signaling Technology, Massachusetts, USA) at 4 °C overnight and it was called Wnt3a-depletion medium.

### Trastuzumab-resistant gastric cancer cell lines

HER-2 expression was detected using western blotting in all seven gastric cancer cell lines, including MKN45, MKN74, SNU216, SNU484, NCI-N87, and AGS. SNU216 and NCI-N87 cells exhibited the highest levels of HER-2 expression (Figure S1a). To simulate the in vivo mode of resistance, we exposed SNU216 and NCI-N87 cells by stepwise exposure to increasing doses of trastuzumab for over a year. Trastuzumab (12 µ g/ml) was added for 48 h during the mitotic phase, and then the cells were transferred into drug-free culture medium until the next mitotic phase (around 10 days). We continued this process while observing cell death every day, changing to fresh complete culture medium, and performing the MTT assay regularly. This process was continued until the concentration of trastuzumab in the medium reached 5000 µ g/ml after 360 days. In the NCI-N87 cell line, we could successfully sub-cultured NCI-N87 cells, which grew steadily in a medium containing trastuzumab, earning the name NCI-N87R. The cells whose final resistance was confirmed were completed around 30 passages, and the numerical value was constantly monitored. However, SNU216 cell growths were not inhibited at all, and cell viability was not decreased even in trastuzumab (5000 µg/ml)-containing medium. As a result, we failed to induce the resistant cell line from SNU216 cells in this study, and it was assumed that they already had trastuzumab-resistance. We observed the trastuzumab resistance of NCI-N87R cells, as revealed by the cell viability assay, while inhibition of trastuzumab on cell viability was seen to increase in a dose-dependent manner in NCI-N87 cells (Figure S1b).

### Cell viability assay

At a density of 3.0-7.5 × 10^3^ cells per well, cells were seeded in 96-well plates, incubated overnight at 37 °C, and then exposed to various concentrations of trastuzumab for 72 h. Each well received a 50 µl aliquot of 3-(4,5-dimethylthiazol-2yl)-2,5-diphenyltetrazolium bromide (MTT) solution (Sigma-Aldrich, St. Louis, MO, USA), and incubation was carried out at 37 °C for an additional 4 h. After removing the medium, 150 µl of dimethyl sulfoxide (DMSO) was added to each well and mixed. A VersaMax microplate reader (Molecular Devices, Sunnyvale, CA, USA) was used to measure the absorbance at 540 nm.

### Spheroid colony formation assay

RPMI 1640 serum-free medium, 20 ng/ml human recombinant basic fibroblast growth factor (Invitrogen), 20 ng/ml human recombinant epidermal growth factor (Invitrogen, Carlsbad, CA, USA), and supplements N2 and B-27 were added to the trypsin-EDTA-isolated NCI-N87 and NCI-N87R cells before they were seeded in each well of an ultralow-attachment 96-well plate (Corning Life Sciences, Acton, MA, USA). Every 4 days, 20 µl of the medium was replaced. Each well was examined under a light microscope after 5, 14, and 21 days, and the size of the spheroidal cells was measured and compared with that of the wild-type cells.

### Western blotting analysis

Whole cells were centrifuged at 15,000 rpm for 10 min at 4 °C after lysis in a RIPA lysis buffer for over 45 min. The supernatant protein concentration was determined using a Bradford assay kit (Bio-Rad Laboratories, Hercules, CA, USA) or a BCA protein assay kit (Thermo scientific, Rockford, MR, USA). Thirty micrograms of denatured protein from each sample were then transferred to a PVDF membrane (Millipore, Billerica, MA, USA) after being separated on a 10% SDS-PAGE gel. The membranes were blocked with 5% skim milk or BSA for 1 h at RT. Next, the membrane was incubated overnight at 4 °C with rabbit anti-polycomb complex protein BMI-1 (BMI1) (1: 500; #ab135713; Abcam, Cambridge, UK), rabbit anti-HER2 (1: 1,000; #2165; Cell Signaling Technology, Danvers, MA, USA), rabbit anti-Snail (1: 500; #3895; Cell Signaling Technology, Massachusetts, USA), rabbit anti-octamer-binding transcription factor 4 (Oct4) (1: 1,000; #2750; Cell Signaling Technology, Massachusetts, USA), rabbit anti Oct4a (1: 500; #2890; Cell Signaling Technology, Massachusetts, USA), and rabbit anti-GAPDH (1: 2,000; #2118; Cell Signaling Technology, Massachusetts, USA) primary antibodies. The membrane was incubated for 1 h at RT with HRP-conjugated anti-rabbit IgG (1:5,000; #7074; Cell Signaling Technology, Massachusetts, USA) secondary antibodies after washing with wash buffer (10 mM Tris-HCl, 70 mM NaCl, and 0.05% Tween 20). The membrane was then washed thrice with wash buffer for 10 min, and ECL (Amersham Biosciences, GE Healthcare, Arlington Heights, IL, USA) was used for detection.

### Immunofluorescence staining of E-Cadherin and β-Catenin

Rabbit monoclonal antibodies against E-cadherin (1:1,000; #sc-7870; Santa Cruz Biotechnology, Dallas, TX, USA) and β-catenin (1:50; #sc-7199; Santa Cruz Biotechnology) were used to label the NCI-N87 and NCI-N87R cells. We observed the cells under a laser-scanning confocal microscope after we stained the nuclei with 1 g/mL DAPI (Sigma-Aldrich, St. Louis, MO, USA) (LSM 780; ZEISS, Oberkochen, Germany).

### Luciferase assay

NCI-N87 and NCI-N87R cells were transfected with pTA-Luc and TCF/LEF luciferase reporter vectors (Promega, Madison, WI, USA). We co-transfected NCI-N87 and NCI-N87R cells with the TopFlash firefly luciferase reporter vector and pRL-SV40-Renilla luciferase vector (Promega). Additionally, we incubated the cells for 72 h to detect the Wnt signaling pathway activity. The dual-luciferase reporter method (Promega) was used to determine the relative luciferase activity.

### Organoid culture

After the study was approved by the ethical committee (IRB 3-2018-0209), clinical samples for organoid establishment and biological analyses were obtained from patients at Gangnam Severance Hospital with informed consent. Gastric cancer specimens were collected via surgical resection or biopsy. Surgical specimens were washed with phosphate-buffered saline (PBS) before being cut into 1-mm^3^ fragments. The fragments were digested with collagenase I (Sigma-Aldrich, St. Louis, MO, USA) at 37 °C for 1 h, and undigested pellets were separated by pressing with a plastic stick. To inactivate the digestive enzymes, the collected epithelia were washed with PBS that was supplemented with 1% bovine serum albumin before performing the plating. For the basal culture medium, advanced Dulbecco’s modified Eagle’s medium/F12 was supplemented with 10 mM HEPES, antibiotic/antimycotic, 1 × B27 supplement (Thermo Fisher Scientific), and 2 mM GlutaMAX. The niche factors were added to the basal culture medium to create a complete medium. The organoids were maintained in an incubator at 37 °C with 5% CO2 and the medium was changed every 3–4 days.

### Statistical analysis

All statistical analyses were conducted using SPSS version 26.0. One-way analysis of variance (ANOVA) was used to determine the difference between the subgroups. A *P* value of less than 0.05 meant that the difference was statistically significant.

## Results

### Trastuzumab-resistant gastric cancer cells exhibit stemness and EMT-like phenotypes

**Fig. 1:**
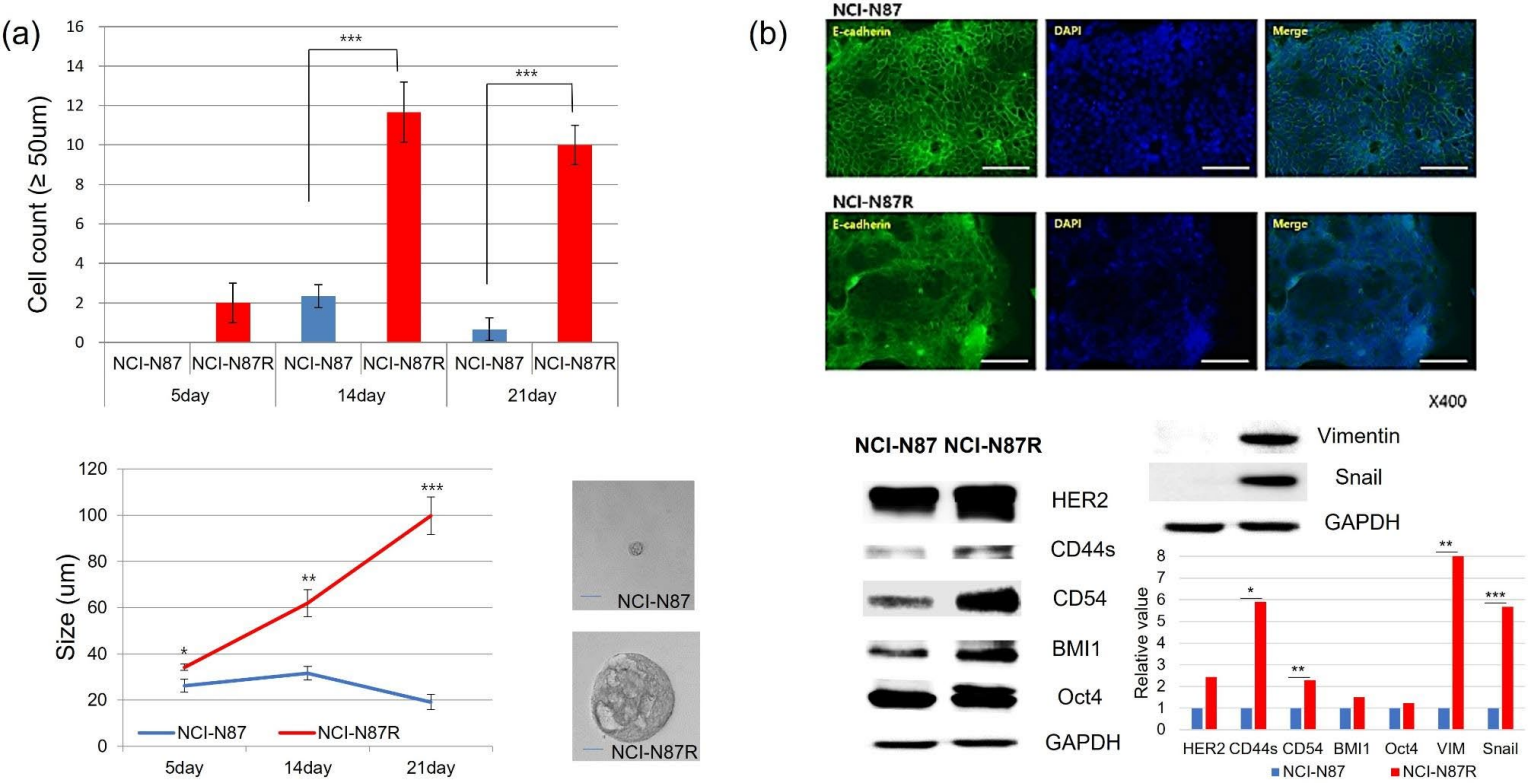
**Stemness and EMT-like phenotypes of trastuzumab-resistant gastric cancer.** (a) NCI-N87R cells had significantly larger sphere size and comprised higher cell counts of spheres larger than 50 μm compared with NCI-N87 cells. (b) E-cadherin was significantly downregulated in NCI-N87R cells, as confirmed by immunofluorescence, compared with parental cells. Stem cell markers including CD44s, CD54, BMI1, Oct4, Vimentin and Snail were significantly upregulated in NCI-N87R cells compared with parental cells, according to Western blot analysis. *P < 0.05, **P < 0.01, ***P < 0.005

To evaluate the stemness of trastuzumab-resistant gastric cancer cells, parental NCI-N87 and NCI-N87R cells were cultured in a suspension for 21 days. Both cell types produced nonadherent spherical colonies known as spheres. Compared with NCI-N87 cells, NCI-N87R cells had significantly larger sphere size with a higher cell count of spheres larger than 50 μm (Fig. 1a). Loss of expression of the epithelial marker E-cadherin is a hallmark of EMT. E-cadherin was significantly downregulated in NCI-N87R cells, as confirmed by immunofluorescence staining. Stem cell markers, including CD44s, CD54, BMI1, OCT4, Vimentin, and Snail, were significantly upregulated in NCI-N87R cells compared with parental cells, as determined by western blot analysis (Fig. 1b). These results suggest that trastuzumab-resistant gastric cancer cells exhibit stemness and overlapping characteristics with EMT cells.

### Trastuzumab-resistant gastric cancer cells exhibit increased activity of wnt signaling pathway

**Fig. 2:**
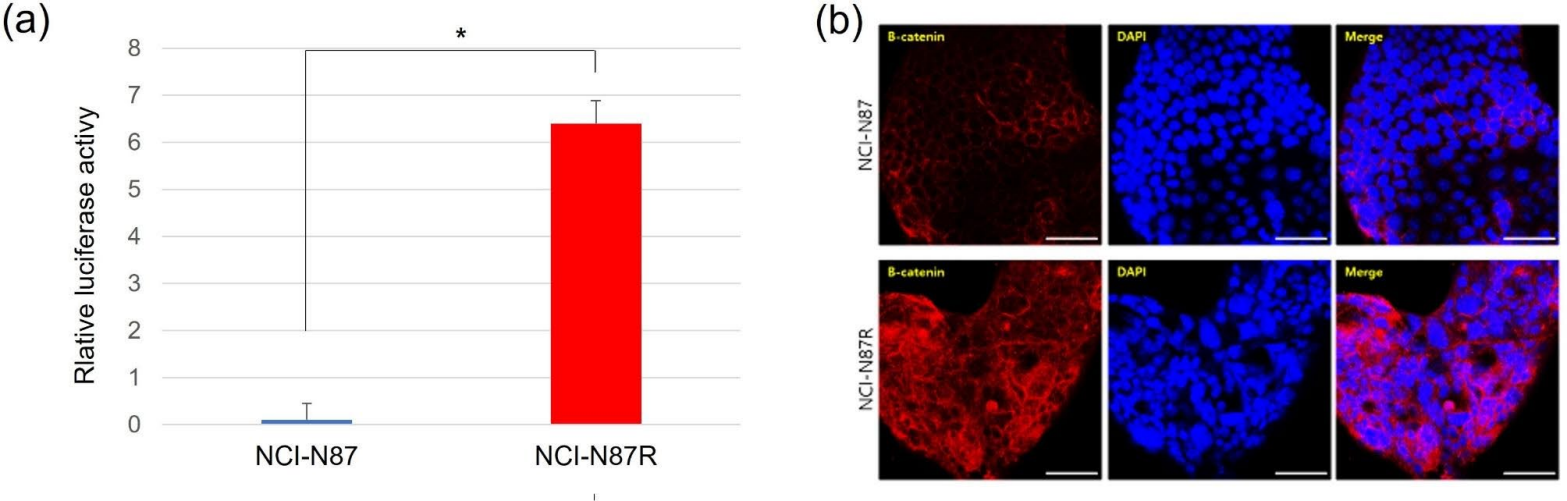
**Increased activity of Wnt signaling pathway of tastuzumab-resistant gastric cancer cells.** (a) NCI-N87R cells showed significantly higher activity of the Wnt signaling pathway compared with that of parental cells. (b) β-catenin was significantly upregulated in NCI-N87R cells compared with parental cells, as observed on immunofluorescence staining. *P* < 0.05, **P < 0.01, ***P < 0.005

As trastuzumab-resistant gastric cancer cells demonstrated EMT-like phenotype changes, we analyzed the activity of the Wnt signaling pathway, which is known to play a crucial role in EMT. The TCF/LEF reporter kit was used to assess the Wnt signaling pathway activity. NCI-N87R cells showed significantly higher activity of the Wnt signaling pathway than parental cells (Fig. 2a). Likewise, immunofluorescence staining showed that β-catenin was significantly upregulated in NCI-N87R cells compared with parental cells (Fig. 2b).

### Gastric cancer cells incubated in Wnt3a-conditioned medium exhibit increased activity of wnt signaling pathway

**Fig. 3:**
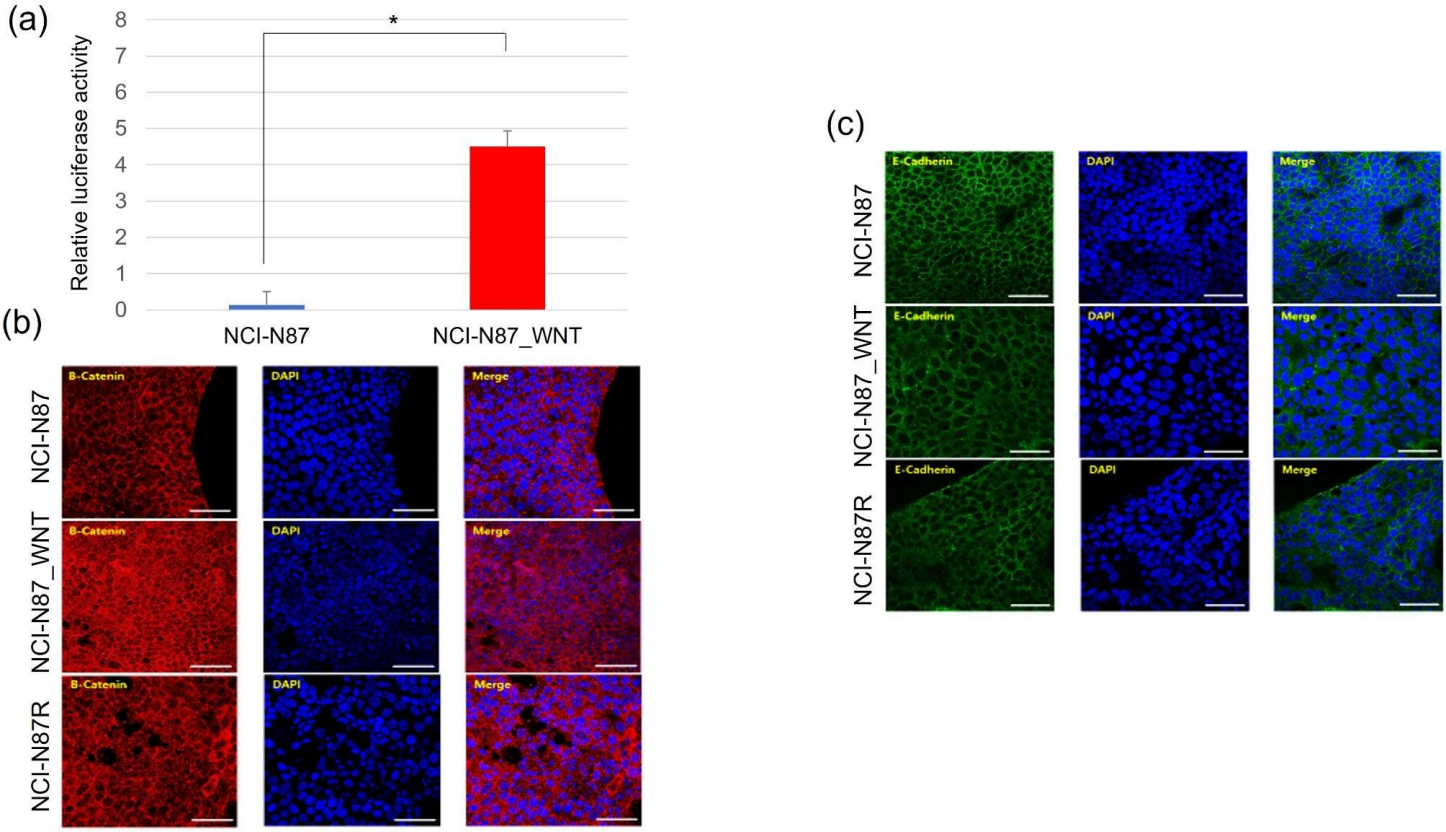
**Increased activity of Wnt signaling pathway of gastric cancer cells incubated in Wnt3a-conditioned medium.** (a) NCI-N87_WNT cells showed significantly increased activity of the Wnt signaling pathway. (b) Immunofluorescence staining revealed that NCI-N87_WNT cells had markedly higher levels of β-catenin expression than NCI-N87 cells. (c) E-cadherin was downregulated in NCI-N87_WNT cells, and this was similar in NCI-N87R cells. *P < 0.05, **P < 0.01, ***P < 0.005

The activity of the Wnt signaling pathway, which plays an important role in EMT, was found to be increased in trastuzumab-resistant gastric cancer cells. Therefore, we investigated whether the Wnt signaling pathway could be activated when parental gastric cancer cells were incubated in the Wnt3a-conditioned medium. NCI-N87 cells incubated in Wnt3a-conditioned medium (NCI-N87_WNT) showed significantly increased activity of the Wnt signaling pathway (Fig. 3a). Immunofluorescence staining revealed that NCI-N87_WNT cells had markedly higher levels of β-catenin expression than NCI-N87 cells (Fig. 3b). Additionally, E-cadherin was downregulated in NCI-N87_WNT cells as well as in NCI-N87R cells (Fig. 3c).

### Gastric cancer cells incubated in Wnt3a-conditioned medium acquired trastuzumab resistance

**Fig. 4:**
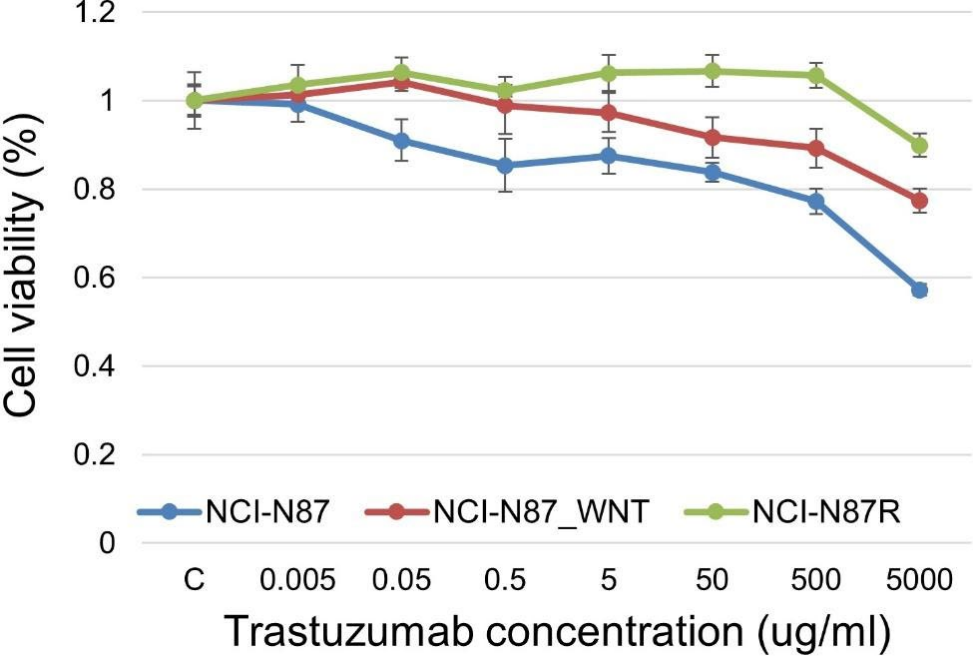
**Acquired trastuzumab resistance of gastric cancer cells incubated in Wnt3a-conditioned medium.** NCI-N87_WNT cells showed lower inhibition of cell proliferation than parental cells

We compared the trastuzumab resistance ability of each cell line using a cell viability assay. NCI-N87_WNT cells showed lower inhibition of cell proliferation than parental cells (Fig. 4).

### Wnt signaling pathway affected trastuzumab resistance of gastric cancer in patients-derived organoids

**Fig. 5:**
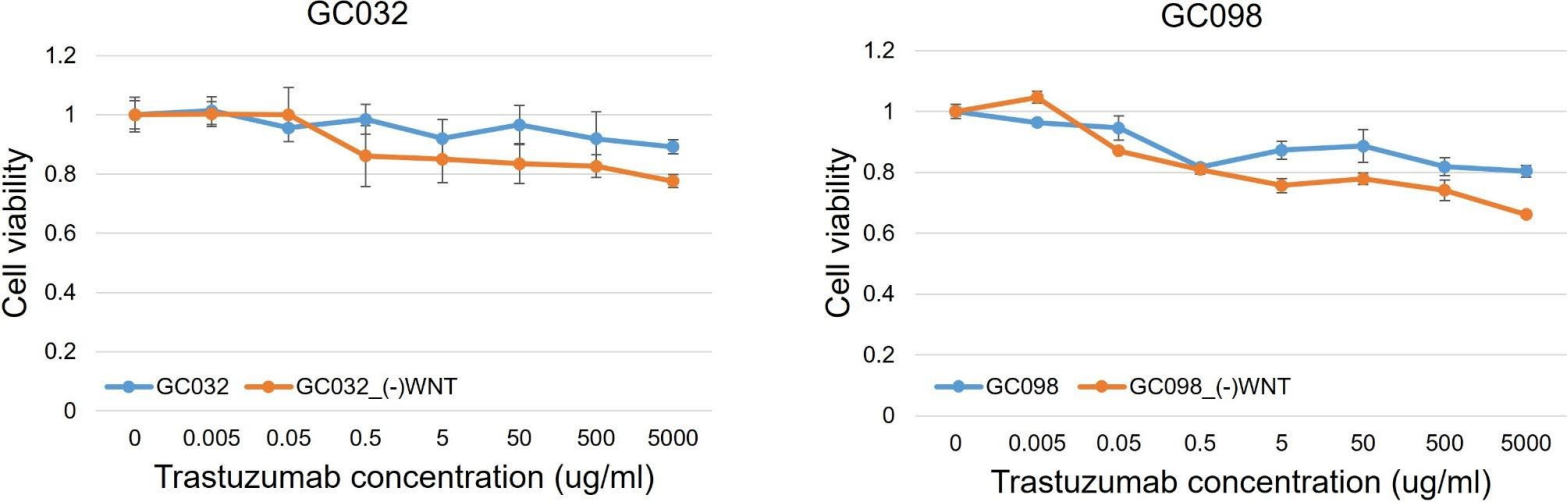
**Trastuzumab resistance of gastric cancer in patients-derived organoids affected by Wnt signaling pathway.** Gastric cancer organoid cells incubated in Wnt3a-depletion medium were more susceptible to dose-dependent inhibition of cell viability by trastuzumab than in complete medium

Next, we investigated whether the Wnt signaling pathway affected trastuzumab resistance on cell viability in a patient-derived organoid. “GC032” and “GC098” are the names of organoid cells derived from gastric cancer patients and cultured in complete medium. “GC032_(-)WNT” and “GC098_(-)WNT” indicate that “GC032” and “GC098” incubated in Wnt3a-depletion medium. We measured the cell viability of each organoid cell by exposing them to escalating doses of trastuzumab. Organoid cells incubated in Wnt3a-depletion medium were more susceptible to dose-dependent inhibition of cell viability by trastuzumab than organoids cultured in complete medium (Fig. 5).

## Discussion

Trastuzumab, one of the most effective anti-HER2 antibodies for breast and gastric cancers, has been used in clinical therapy for a long time; however, the emergence of resistance is a significant barrier to trastuzumab-based treatment for HER2-overexpressing breast and gastric cancers. Although numerous mechanisms of trastuzumab resistance have been suggested in association with breast cancer, it is unclear whether the same mechanism applies for gastric cancer. Therefore, it is crucial to understand the mechanisms and identify the phenotype of trastuzumab resistance in gastric cancer to develop novel therapeutic approaches.

In the current study, trastuzumab-resistant cells were obtained in vitro from the human gastric cancer cell lines NCI-N87 through repeated, dose-escalating trastuzumab treatment. Trastuzumab-resistant gastric cancer cells showed higher levels of stemness and EMT characteristics along with obvious acquisition of mesenchymal morphology, decreased levels of epithelial markers, and increased levels of mesenchymal markers. Self-renewal (forming spheres), increased clonogenicity, and tumorigenicity were also observed in trastuzumab-resistant gastric cancer cells. EMT can trigger reversion to a CSC. CSCs, which can initiate tumorigenesis and have high metastatic potential, frequently develop resistance to chemotherapeutic agents [20]. The combination of stemness and EMT is an independent predictor of outcomes in patients with gastric cancer [23]. It was recently shown that ectopic expression of the embryonic stem cells transcription factor, NANOGP8, in gastric cancer cells, promotes sphere formation and chemo-resistance by up-regulating EMT inducers and CSCs markers [24]. Furthermore, drug resistance is substantially correlated with the expression of LGR5 and EMT-related genes in gastric cancer sphere cells [25]. These findings suggest that prolonged trastuzumab treatment induces stemness and EMT-like phenotype in gastric cancer cells, leading to trastuzumab resistance.

Through EMT induction, pathophysiological conditions, such as tissue damage or tumorigenesis, can cause differentiated cells to develop a multipotent stem cell-like phenotype. This process is similar to developmentally regulated EMT signaling pathways, such as Wnt, Notch, and Hedgehog, which are responsible for both normal and CSC renewal and maintenance [26, 27]. Wnt is a highly conserved signaling pathway that regulates embryonic and organ development as well as the progression of various types of human cancers, such as breast cancer, ovarian cancer, colorectal cancer, and prostate cancer [28]. Recent genome-wide sequencing and gene expression profile analyses have revealed that Wnt signaling is primarily involved in cancer proliferation and metastasis. Recent studies have revealed that Wnt signaling is essential for breast cancer immune microenvironment regulation, stemness maintenance, therapeutic resistance, and phenotype shaping [29–31]. Wu et al. showed that Wnt3 overexpression in breast cancer cells resistant to trastuzumab activates the Wnt signaling pathway, which induces transactivation of EGFR and promotes EMT-like transition [16]. The EMT-like transition in cancer cells may promote tumor invasion, metastases, and drug resistance. Data from our current study indicate that trastuzumab-resistant gastric cancer cells exhibit increased activity of the Wnt signaling pathway. Additionally gastric cancer cells incubated in Wnt3a-conditioned medium exhibit increased activity of Wnt signaling pathway and trastuzumab resistance. We also investigated how the Wnt signaling pathway affected trastuzumab resistance on cell viability in a patient-derived preclinical model. Organoid cells incubated in Wnt3a-depletion medium were more susceptible to dose-dependent inhibition of cell viability by trastuzumab than the parental cells.

In several recent studies, it was importantly noted that EMT-related signaling interacts functionally with the autophagy pathway, which is intricately linked to the fate of cancer cells [32]. Interestingly, autophagy also exhibits a dual effect on EMT, depending on the setting, either activating or inhibiting it. Through modulating autophagy signaling pathways, such as integrin, NF-κB, Wnt and TGF-β, the EMT process has a dramatic effect on autophagy modulation [33]. Given the complexity of this interaction, elucidating the mechanisms of their mutual regulation is challenging, but have clinical benefits in cancer treatment.

This study has limitations that need to be addressed. First, we could obtain only one resistant cell line of NCI-N87R. When we exposed SNU216 and NCI-N87 cells to trastuzumab, SNU 216 cells were unable to produce resistant cells and it was consequently assumed that the SNU 216 cell line already exhibited trastuzumab-resistance. In addition, knock-down or knock-out of Wnt3a study is required to ascertain whether the Wnt/β-catenin pathway is critical for maintenance of trastuzumab resistance.

Our study revealed that trastuzumab-resistant gastric cancer cells exhibit EMT-like phenotype by promoting the Wnt signaling pathway. Since cancers are heterogeneous, future efforts to find new treatment options that target the plasticity of cancer cells and improve survival outcomes should be explored. EMT induction and the emergence of CSCs are associated with plasticity and drug resistance. Several key signaling pathways, including Wnt, which are known inducers of EMT and promoters of stem cell maintenance, contribute to this process [21]. We conclude that the Wnt signaling pathway may be a target for trastuzumab-resistant gastric cancer, and that Wnt signaling pathway inhibitors combined with trastuzumab may provide a promising treatment strategy for patients with trastuzumab-refractory gastric cancer. Further research is required to understand the trastuzumab-resistant mechanism for an individualized and precise treatment of gastric cancer.

### Electronic supplementary material

Below is the link to the electronic supplementary material.

Supplementary Material 1

Supplementary Material 2

## Data Availability

The data that support the findings of this study are available from the corresponding author upon reasonable request.
