# Supplementary figures and images for: Wnt/β-catenin pathway is a key signaling pathway to trastuzumab resistance in gastric cancer cells
Source: BMC Cancer. 2023 Sep 29;23:922. doi: 10.1186/s12885-023-11447-4 (PMC10542239; doi:10.1186/s12885-023-11447-4)

# Image quant Las4000

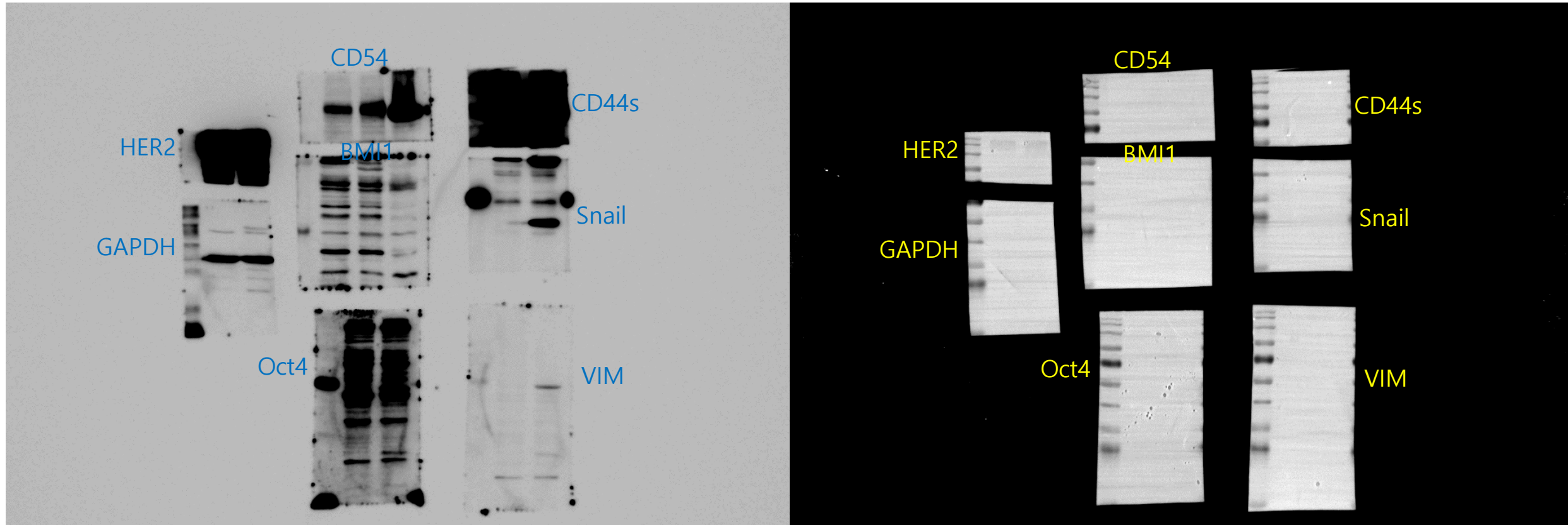

# Film develop

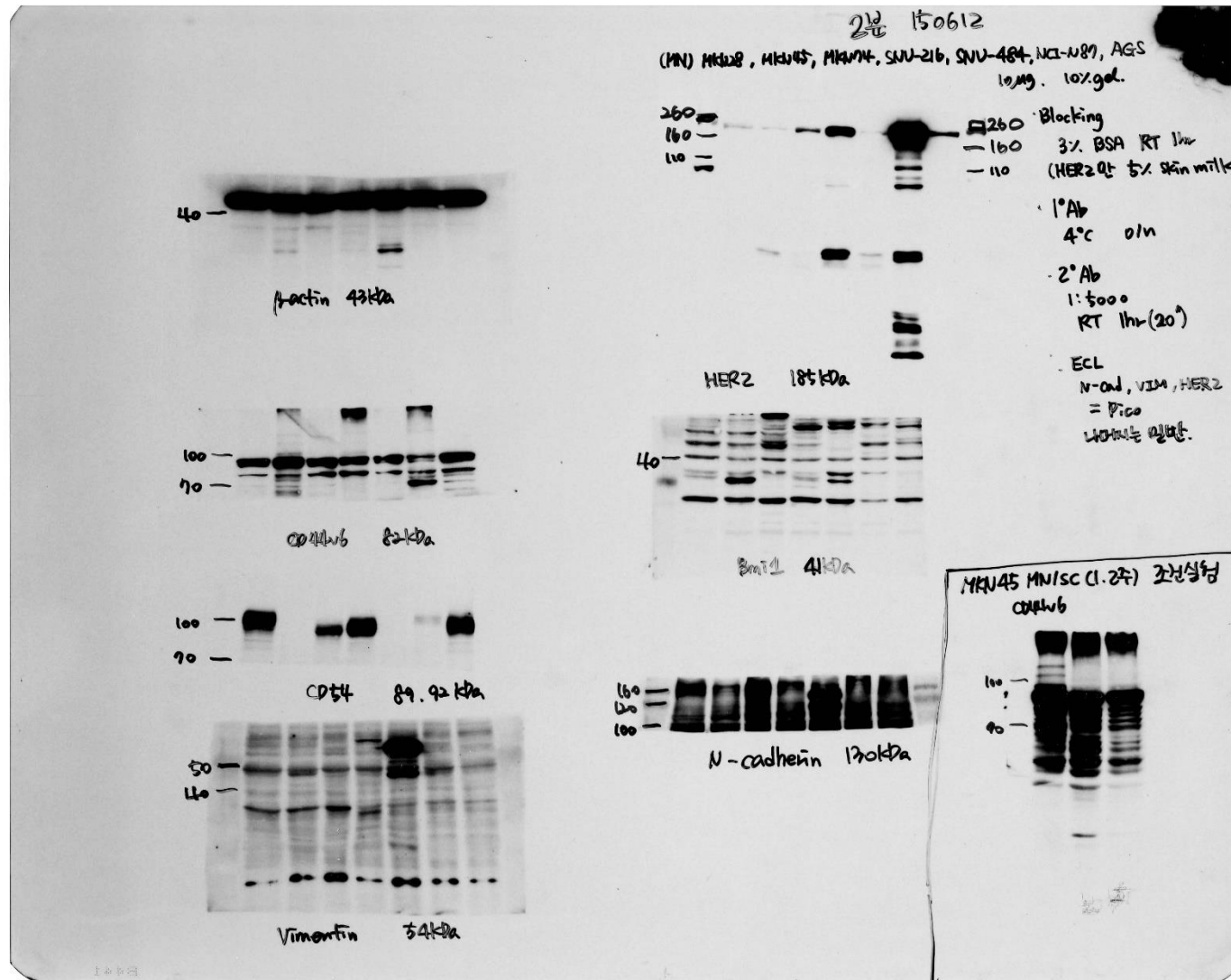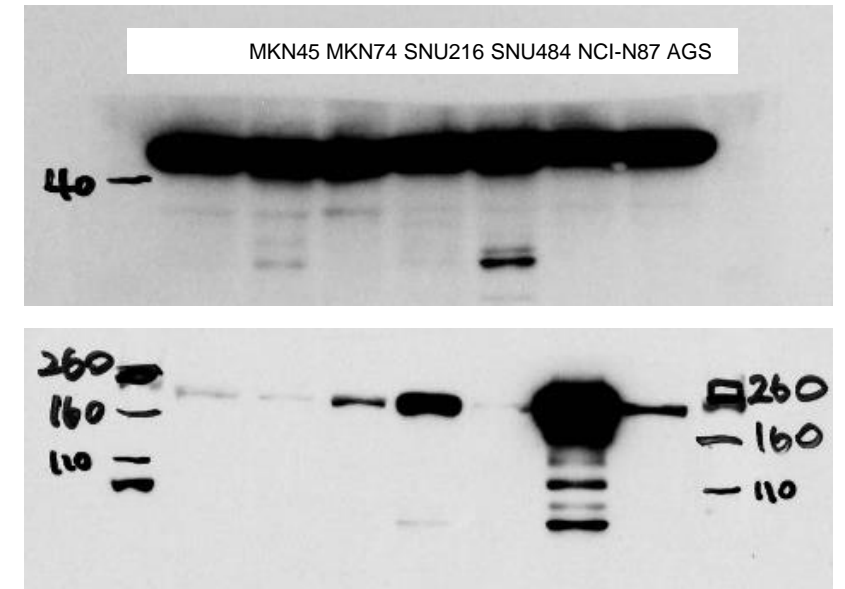

Supplement: Supplementary file 1 — Supplementary Material 1 [file 12885_2023_11447_MOESM1_ESM.pdf]

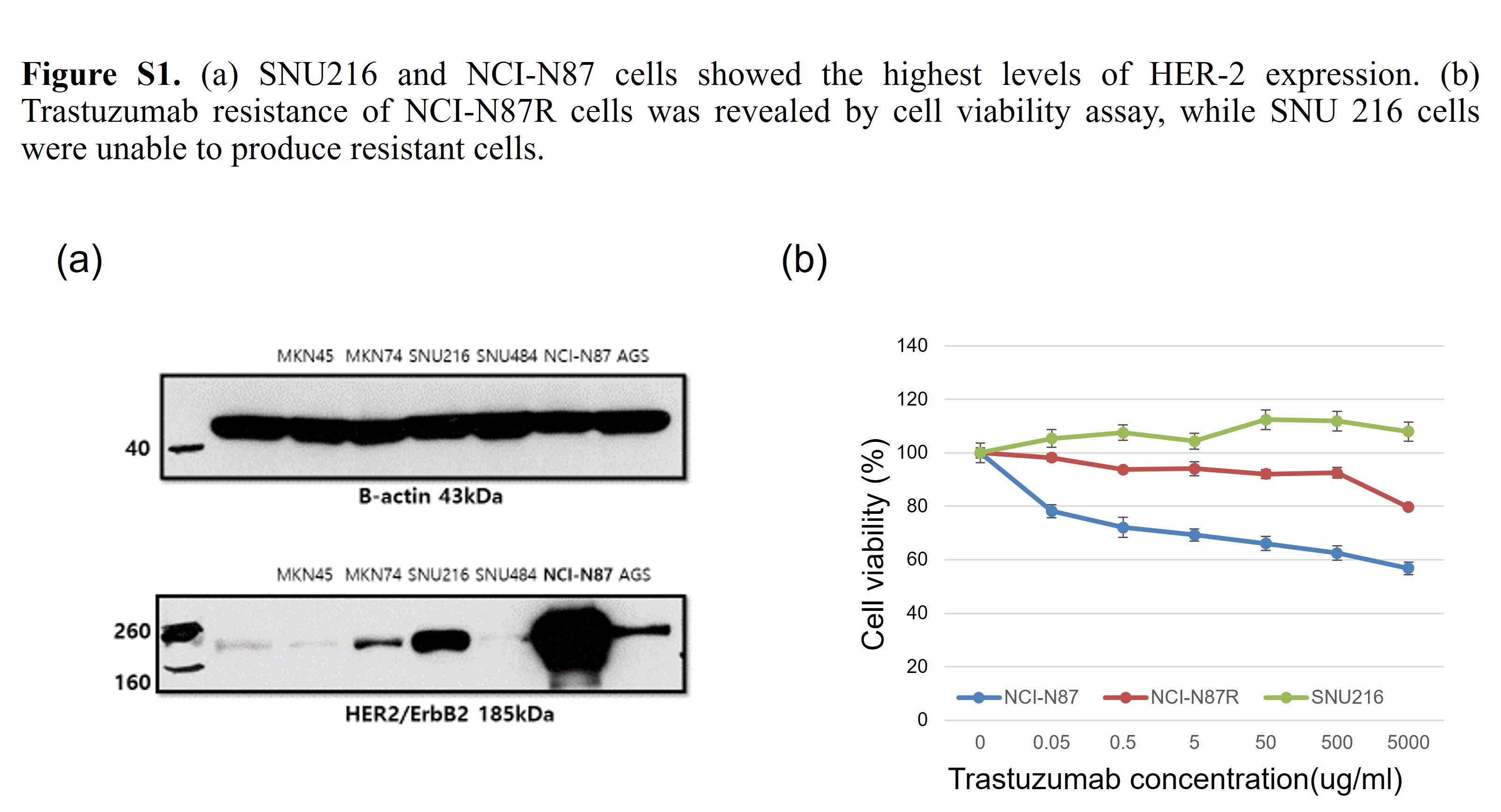

Supplement: Supplementary file 2 — Supplementary Material 2 [file 12885_2023_11447_MOESM2_ESM.jpg]
